# Radiographic change over 11 years in a patient with asbestos‐related pleural disease

**DOI:** 10.1002/rcr2.642

**Published:** 2020-08-19

**Authors:** Reina Hara, Yukihiro Yano, Fukuko Okabe, Tomoki Kuge, Masahide Mori, Koji Urasaki

**Affiliations:** ^1^ Department of Thoracic Oncology Osaka Toneyama Medical Center Osaka Japan; ^2^ Department of Respiratory Medicine and Clinical Immunology, Graduate School of Medicine Osaka University Osaka Japan; ^3^ Department of Pathology Osaka Toneyama Medical Center Osaka Japan

**Keywords:** Asbestos body, autopsy, benign asbestos pleural effusion, diffuse pleural thickening, radiographic change

## Abstract

Asbestos‐related pulmonary conditions such as benign asbestos pleural effusion (BAPE) and diffuse pleural thickening (DPT) can develop after many years of asbestos exposure. These conditions cause progressing constrictive deficit in pulmonary function which may lead to respiratory failure and death. We report the case of a 72‐year‐old man with asbestos‐related BAPE and DPT which developed approximately 40 years after occupational asbestos exposure, leading to chronic respiratory failure and death. We were able to observe his clinical course including computed tomography (CT) scan evaluation over 11 years. In addition to this observation, moderate asbestos body concentration was confirmed in autopsy‐derived lung tissue. There are few case reports that showed radiographic course of asbestos‐related pulmonary disorder initiated as BAPE, followed by unilateral DPT and later bilateral DPT that was histologically proven with asbestos body. We consider his clinical course is important in managing this disease, especially in early phase.

## Graphical abstract

We report the case of a 72‐year‐old man with asbestos‐related disease, firstly benign asbestos pleural effusion and subsequently diffuse pleural thickening, which progressed over 11 years and finally presented chronic respiratory failure. After his death, moderate asbestos body concentration was proven in his autopsy‐derived lung tissue.

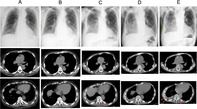

## Introduction

Asbestos‐related pulmonary conditions include pleural plaques, benign asbestos pleural effusion (BAPE), diffuse pleural thickening (DPT), asbestosis, malignant mesothelioma, and bronchogenic carcinoma. BAPEs are the earliest pleural‐based manifestation which usually occur within 10 years of exposure, sometimes after decades. DPT refers to extensive fibrosis of the visceral pleura caused by asbestos exhibiting continuous pleural thickening. These conditions are known to cause progressing constrictive deficit in pulmonary function which may lead to respiratory failure and death [1]. Diagnosis of both asbestos‐related BAPE and DPT is made based on a history of asbestos exposure and radiological findings after exclusion of other pleural effusions: most importantly tuberculous pleuritis, bacterial pleuritis, collagen diseases, and malignant diseases including malignant mesothelioma. When asbestos‐related diseases are suspected, asbestos fibre/body concentration level in lung tissues can be utilized in making clinical judgement.

We report the case of a 72‐year‐old man with asbestos‐related disease, firstly BAPE and subsequently DPT, which progressed over 11 years and finally presented chronic respiratory failure. After his death, moderate asbestos body concentration was proven in his autopsy‐derived lung tissue.

## Case Report

**Figure 1:**
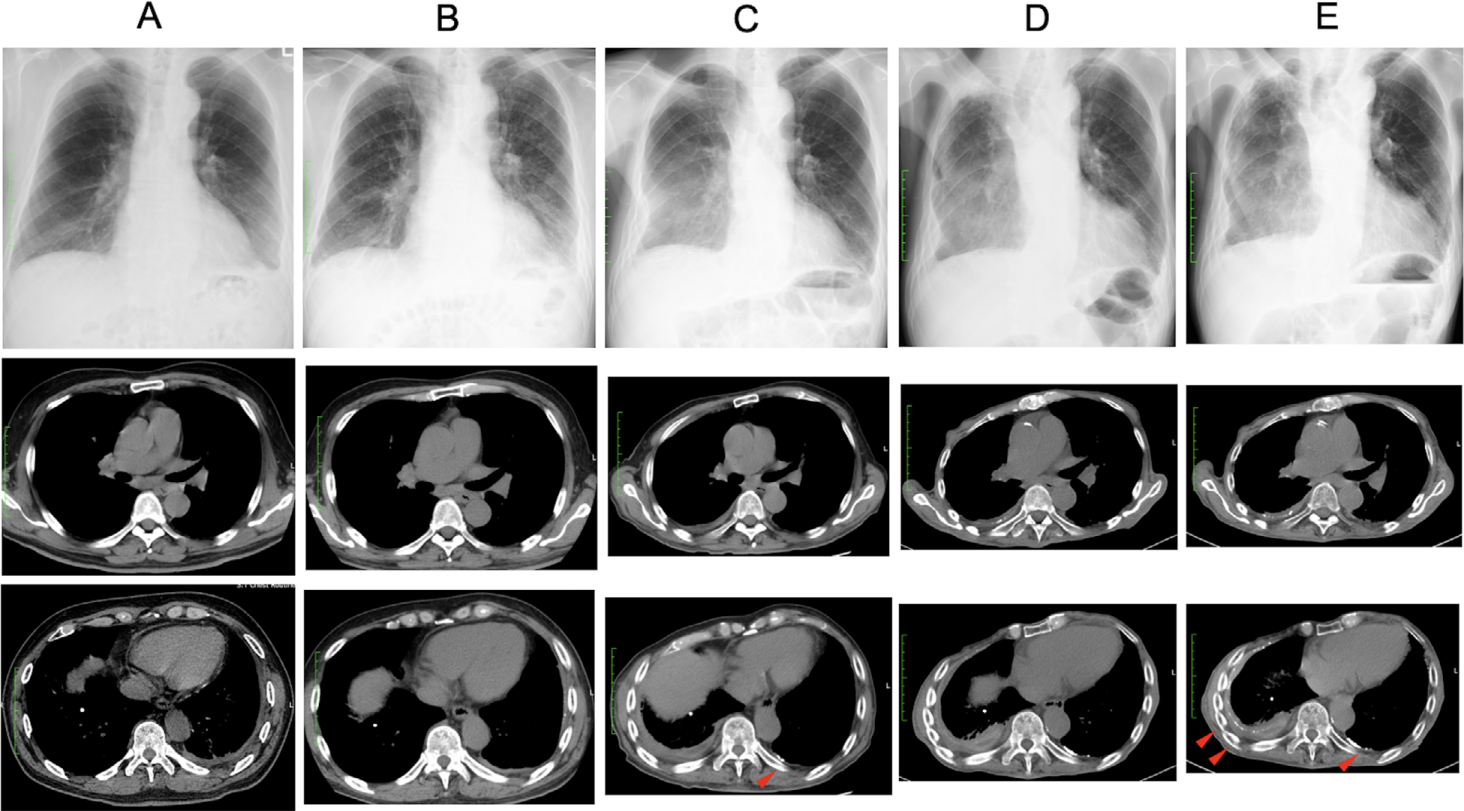
Chest X‐ray and chest computed tomography (CT) six years before (A), three years before (B), and on the day of the first visit (C). X‐ray: Costophrenic angle blunting is observed first on the left (A, B) and then on the right (C). CT: Left pleural effusion is replaced by pleural thickening with calcifications (C, arrow). Chest X‐ray and chest CT on the 44th (D) and 56th (E) months from the day of the first visit. X‐ray: The size of the right lung is declined gradually over 11 years (D, E). CT: Both right and left diffuse pleural thickening with calcifications show progression (E,arrows).

A 72‐year‐old man was referred to our hospital for suspected right pulmonary effusion on chest X‐ray. For approximately three years around the age of 30, he dealt with serpentine asbestos in research. Seven years before he was referred to our hospital, he visited another hospital for suspected left pleural effusion on chest X‐ray, and his chest computed tomography (CT) showed left pleural effusion and ipsilateral pleural thickening; after two years of follow‐up, the pleural effusion had resolved without treatment (Fig. 1A). Two years later, he had similar abnormalities pointed out on chest X‐ray, and his chest CT revealed unchanged left pleural thickening; with no apparent change, his follow‐up was finished after one year (Fig. 1B).

At the first visit to our hospital, there were no specific findings on physical examination as well as laboratory tests, except for slightly low serum haemoglobin level. Chest CT showed bilateral pleural thickening accompanied with calcification on the left pleura (Fig. 1C). Over the next four years, thickening and calcification of the pleura showed gradual progression, and accordingly his lung volumes declined (Fig. 1D).

**Figure 2:**
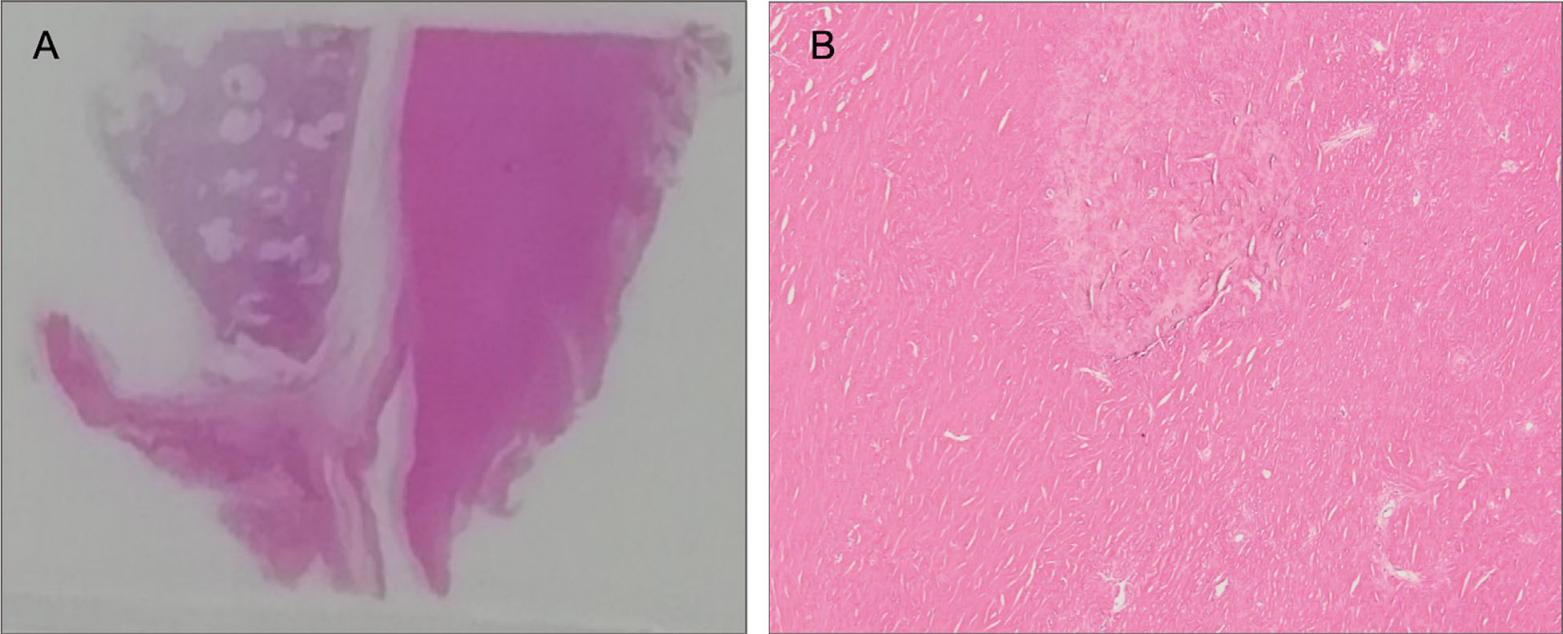
Histopathological features of autopsy‐derived lung tissue obtained from left S9 region with haematoxylin and eosin stain. The membranes around both lungs consist of hyalinized fibrous tissue with patchy infiltration of lymphocytes. These findings are consistent with diffuse pleural thickening. No obvious asbestos body is detected. (A: loupe image, B: 400× magnification).

During this progression, he was admitted to our hospital for acute respiratory failure caused by bacterial pneumonia. Despite resolution of pneumonia, he developed hypercapnic chronic respiratory failure, and therefore home oxygen therapy and non‐invasive positive pressure ventilation (NPPV) were initiated. Subsequently, he experienced recurrent bacterial pneumonia, and 53 months since the first visit he was again hospitalized for acute‐on‐chronic respiratory failure. Thickening and calcification of the pleura progressed furthermore (Fig. 1E). Despite treatment, he died on the 94th day of admission; autopsy was performed with the consent of his family. On gross finding, the bilateral lungs were entirely covered with white, hard, and fibrous membrane and adhered to the chest wall. Microscopically, the membrane consisted of hyalinized fibrous tissue with patchy infiltration of lymphocytes. No obvious asbestos body was detected. (Fig. 2) Asbestos body concentration was measured at the Kobe Rosai Hospital. Tissue samples were derived from the right lower lobe and processed using standard methods. Asbestos body concentration was 1086/g dry lung tissue, indicating moderate asbestos exposure.

## Discussion

In the present case, we were able to observe clinical course of asbestos‐related disease over 11 years, initiated as BAPE, followed by unilateral DPT and later bilateral DPT. He suffered from chronic respiratory failure due to constrictive deficit in DPT and later died.

Fujimoto et al. reported that seven patients out of 70 patients died due to respiratory failure with BAPE [2]. Jeebun et al. reported 75 cases with DPT that were assessed over 15 years. In this report, 72% of cases had unilateral disease at presentation and 24% were observed to develop contralateral disease after a median of two years. In 40%, DPT was preceded by the development of BAPE and these effusions were seen to resolve and recur [3]. There are limited number of reports that showed the radiographic change from BAPE or DPT to massive pleural thickening that result in respiratory failure. Gocho et al. reported a case of intractable BAPE leading to remarkable restrictive ventilator impairment [4]. Other than this case report, we could not find any report that showed radiographic course of patient who has progressive BAPE or DPT. We show radiographic course of the present case and decline of respiratory conditions with progression of DPT is considered to indicate the involvement of constrictive deficit.

Asbestos fibre/body concentration in lung tissues is useful in evaluating the past exposure to asbestos. Although very few data described the correlation between asbestos fibre/body levels in various asbestos‐associated diseases, the levels in asbestos‐related DPT are generally higher than in pleural plaques cases but lower than in asbestosis cases [5]. From research on 255 lung cancer patients, Kohyama classified the estimated past asbestos exposures according to asbestos body concentration as follows: low if less than 1000/g dry lung tissue, intermediate if between 1000 and 5000/g dry lung tissue, and high if exceeding 5000/g dry lung tissue [6]. He considered low‐, intermediate‐, and high‐exposure patients as general population, intermediate exposure risk population, and occupational exposure risk population, respectively. The present case belongs to “intermediate,” and together with the past occupational exposures the pulmonary manifestations were judged as asbestos‐related.

In conclusion, we were able to observe radiographic change over 11 years of asbestos‐related pulmonary disease in an autopsied case. We consider his clinical course is important in managing this disease, especially in early phase.

### Disclosure Statement

Appropriate written informed consent was obtained for publication of this case report and accompanying images.
